# Antibacterial Activity of Isolated Immunodominant Proteins of *Naja Naja (Oxiana)* Venom

**Published:** 2017

**Authors:** Mahboobeh talebi mehrdar, Rasool madani, Reza hajihosseini, Soheila moradi bidhendi

**Affiliations:** a *Department of Biochemistry, Payame Noor university,19395-4697, Tehran, Iran. *; b *Proteomics &Biotechnology department Razi Institute. *; c *Department of Biochemistry, Payame Noor university,19395-4697, Tehran, Iran. *; d *Microbiology department, Razi Vaccine and Serum Research Institute, Agricultural Research, Education and Extension Organization , Karaj, Alborz. PC. 3197619751. Iran.*

**Keywords:** *Naja Naja*, Immunodominant protein, Antibacterial effect, Electroelution

## Abstract

The aim of this study is to investigate antibacterial effects of immunodominant proteins isolated from the venom of *Naja Naja Oxiana *snake against *Staphylococcus aureus, Escherichia coli, Bacillus subtilis *and *Pseudomonas aeruginosa.* The innate immune system is an important line of defense against bacterial diseases. Antibacterial peptides and proteins produced by snake venoms have recently attracted significant attention due to their relevance to bacterial diseases and the potential of being converted into new therapeutic agents. Identification of immunodominant proteins of the venom of *Naja Naja Oxiana* snake was performed by SDS-PAGE and western blot analysis. Identified proteins were isolated directly from preparative gel electrophoresis by Electro-elution. In the next step, antibacterial effects of immunodominant proteins were tested against several strains of clinical isolates, including *S.aureus, B.subtilis (*Gram-positive bacteria) *P.aeruginosa *and *E.coli* (Gram-negative bacteria) using broth microdilution and disc-diffusion assays. In order to compare the results of the disc-diffusion assay, antibacterial effects of several antibiotics (Gentamicin, Ampicillin, Penicillin, Amoxicillin and Ciprofloxacin) were also examined using the same conditions.

Results showed that immunodominant proteins of (14, and 65kDa) with high immunogenicity were very effective in inhibiting the growth of two Gram-positive bacteria (*S.aureus, B.sub*) that were tested. However, they were only moderately effective in inhibiting the growth of the two tested Gram-negative bacteria (*P.aeruginosa *and *E.coli*). However, immunodominant proteins of 22 kDa and 32kDa with high immunogenicity, showed slight effectiveness in inhibiting the growth of two; the Gram-positive and Gram-negative bacteria that were tested. To the best of our knowledge, these immunodominant proteins are novel antigens for potent antimicrobial effects against two gram-positive bacteria (*S.aureus, B.subtilis* ) and less antimicrobial effect against two gram-negative bacteria (*E.coli, P.aeruginosa*) that were prepared .

## Introduction

Snake venom is a complex mixture of peptides, proteins, different enzymes including phospholipases_,_ oxidases, nucleosidase, ribonuclease, carbohydrate, and mineral that contain a variety of chemicals namely pharmacological and toxicological proteins. Antibacterial and anticancer peptides and proteins produced by snake venom have recently attracted attention for the purpose of identifying new therapeutic agents. The first reports about antibacterial effects of snake venoms were documented (1948 and 1968) for Elapidae and Viperidae (1, 2). Snake venoms of the Viperidae family have antibacterial effects against *Sarcina *species, while a lytic factor or cytotoxin found in *Naja *species, was composed of a basic low-molecular-weight protein. Also *Hemachatus haemachatus *venom has been shown to have antibacterial activity; these venoms kill *Escherichia coli* (3). Several antibacterial studies on Crotalidae (4, 5), Elapidae and Viperidae (6, 7) snake venoms have also been mentioned in the literature to have antibacterial activity against Gram-negative and Gram-positive bacteria. In addition, several studies have reported the antibacterial effects of proteins extracted from *Pseudechis australis *(Australian King Brown) (7).

Accordingly, previous studies have demonstrated that L-amino acid oxidase (8, 9), lectin (10) and phospholipase A_2_ (11) have antibacterial components. Antimicrobial agents inhibit the growth of microorganisms at low concentrations. These compounds include antibiotics which are naturally occurring substances from venoms and microorganisms, as well as chemically-synthesized substances. Therefore, antibiotics that are produced from venoms and microorganisms can be used to treat bacterial diseases instead of chemotherapeutics (12). Antibacterial proteins are widely expressed in snake venoms (13, 14). A variety of snake venom proteins and peptides have been investigated in previous studies and they have demonstrated that these antibacterial proteins and peptides are highly active in killing a wide range of bacteria (14-16). In the present study, the antibacterial effect of isolated immunodominant proteins (14, 22, 32, and 65kDa) from the venom* of Naja Naja Oxiana* snake was tested against several strains of clinical isolates including *S.aureus, B.subtilis (*Gram-positive bacteria) *P.aeruginosa,* and* E.coli* (Gram-negative bacteria) using broth microdilution and disc-diffusion assays.

## Materials and Methods

Lyophilized crude venom of *Naja Naja (Oxiana)* was a kind gift from poisonous animal Dept.Razi Vaccine and serum research Institute, Karaj –Iran.

The serum of hyper-immunized horses was provided by Razi Institute, Karaj-Iran

Rabbit Anti-horse – IgG (whole molecule) – peroxidase conjugate, was prepared from sigma.

*Micro-organisms*

The Gram-positive bacteria including *Bacillus subtilis* (ATCC 6633) and* Staphylococcus aureus* (ATCC 6538) and Gram-negative bacteria including *Escherichia coli (*ATCC 25922) and *Pseudomonas aeruginosa (*ATCC 97853) were obtained from RTCC (Razi Type Culture Collection)

*Standard Antibiotics*

Ampicillin (10 μg), Gentamicin (10 μg), Penicillin (10 μg), Ciprofloxacin (5 μg), and Amoxiclav (25 μg)

*Chemical Reagents*

Ammonium persulfate (APS), Tween 20, Acrylamide, SDS and TEMED were obtained from Bio-Rad (USA); phosphate buffered salt (PBS) was acquired from Merck, Bovine serum albumin (BSA), chloro-1-naphthol from Fluka, and hydrogen peroxidas were obtained from Sigma.

*Determination of protein concentration*

Protein concentration was determined by the method of Lowry using BSA as standard (17).

*SDS-PAGE*

Electrophoresis was carried out on a 15% polyacrylamide gel by the method of laemmli (18). Crude venom (20 μg) was mixed with an equal volume of sample buffer and molecular weight marker separately loaded into the wells. The wells were filled with running gel buffer. The output voltage was 110V for 75 min. After the run, the gel was stained with Coomassie Brilliant Blue R-250 (sigma) and then it was distained. Molecular weights of proteins of crude venom were estimated according to the bands pattern of obtained marker proteins.

*Western Blotting*

The proteins of venom were denatured, separated on 15% SDS-PAGE gel, and transferred onto nitrocellulose membrane (sigma) by semi-dry transfer cell (Bio-Rad, Hercules, CA). The membrane was blocked with 3% bovine serum albumin (BSA) and incubated at room temperature for 90 min. The membrane was washed with several changes of PBST (phosphate buffered salt tween) buffer and incubated with diluted horse antivenom solution (1:20 diluted with PBST buffer) at 4 °C overnight. Then the membrane was washed with several changes of PBST buffer and incubated with appropriate HRP-conjugated rabbit anti-horse secondary antibody (1:10,000 dilution with PBST buffer) for 90 min at room temperature. After washing off unbound secondary antibody with PBST buffer, the bonds were visualized with substrate buffer (18 mg 4-chloro-1-naphthol (Fluka)), 24 mL PBS and 3 μL hydrogen peroxidase (4).

*Isolation of immunodominant proteins identified by Western Blot Electro-elution*

In the present study, Bio-Rad model 422 electro-eluter was used to recover 4 sharp bond immunodominant proteins that were identified by western blot and separated by SDS-PAGE electrophoresis.

Briefly, after running a standard 15% preparative gel electrophoresis, the gel was stained with Coomassie Blue to visualize the protein bands. The expected (14, 22, 32, and 65kDa) protein bands were cut out and diced into smaller slices and placed into 6 tubes. The tubes were filled with elution buffer (Tris/Glycin buffer was also used for gel electrophoresis), then, silicone adaptors were filled with elution buffer and checked for leakage and air bubbles; then, the lead was attached and the elution conditions were set (8 to 10 mA/glass tube) for 5h, on a magnetic stirrer.

The per chamber was removed and the eluted proteins were pipetted from the membrane. Next, they were dialyzed in elution buffer without SDS (overnight at 4 °C) in order to remove SDS from isolated proteins; then it was concentrated with PEG.

*Disc Diffusion*

Disc-diffusion assay was performed by the published method (Bauer *et al.*, 1966). Pure cultures were prepared by sub culturing the test strain into 10 mL of Brain Heart Infusion broth (BHI broth) (Merck), following incubation at 37 °C for 24 h. The culture media was diluted and adjusted to 0.5 McFarland standard, containing 1.5x10^8^ cfu ml ^-1^, in order to inoculate the same dose of bacteria when repeating the experiment. The absorbance of the cultured media was also determined at 600 nm, using a spectrophotometer apparatus (Jenway 6105, Essex, England).

Sterile blank paper discs (7 mm in diameter) were then placed on MH agar surface and 40 µL of isolated immunodominant proteins (14, 22, 32, and 65 kDa) were added per disc in four replicates. Antibiogram discs including, Ampicillin (10 μg), Gentamicin (10 μg), Penicillin (10 μg), Ciprofloxacin (5 μg), and Amoxiclav (25 μg) were used as positive controls. The plates were incubated at 37 °C for 16-18h and the zones of inhibition were measured. The experiments were performed at least in four replicates.

*Broth Microdilution Assay*

Broth microdilution assay was determined according to the protocol explained by the NCCLS, National Committee for Clinical Laboratory Standards (2000). 

Bacteria suspensions were cultured in Muller-Hinton broth at 37 °C. The inoculum suspension was adjusted to the density of 0.5 McFarland (1.5× 10^8^ cfu/mL). Bacterial suspensions (50 μL) were incubated in a 96-well plate in the presence of 50 μL of immunodominant proteins (14kDa ranging from 600 to 4.68 μg/mL), 22 kDa ranging from 380 to 2.96 μg/mL, 32kDa ranging from 350 to 2.68 μg/mL and 65 kDa from 500 to 3.87 μg/mL, using serial dilutions. The plates were incubated at 37 °C for16 -18 h. The MIC was defined as the lowest concentration of protein that visibly inhibited bacterial growth (two assays for each). The plates were read at 620 nm in a microplate reader (Epoch-Bio TeK) after being incubated overnight (~ 16 h) at 37 °C.

## Results

*Determination of Protein Concentration*

Protein concentration of crude *Naja Naja* venom was determined 16 mg/mL by BSA standard protein using the Lowry method.

*SDS-PAGE*

**Figure 1:**
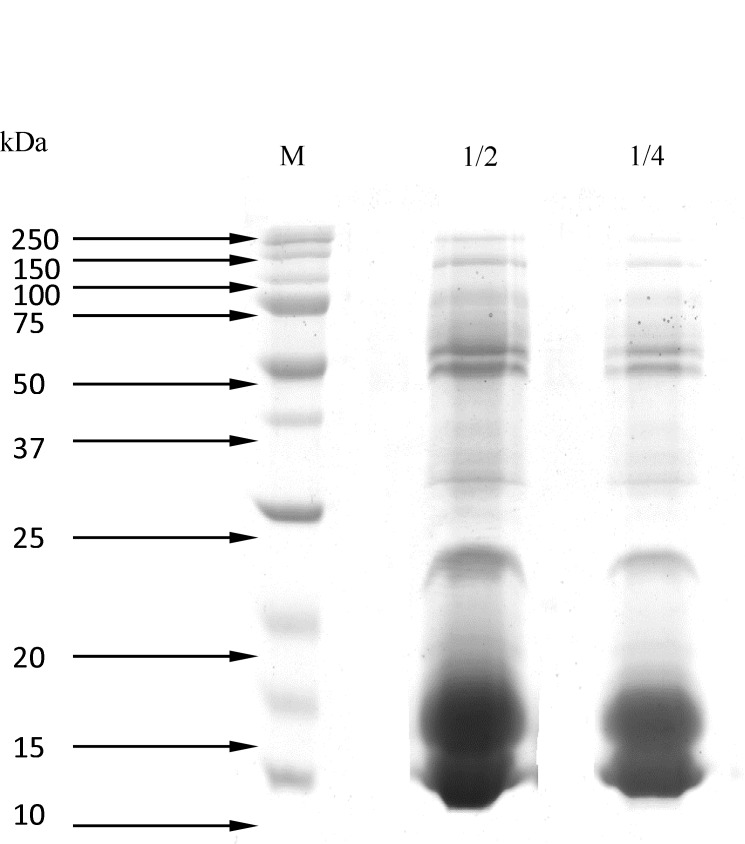
Separation of *Naja Naja* proteins venom by SDS-PAGE (15%) 1) Molecular weight markers. 2) crude venom (dilution 1:2).3) crude venom (dilution 1:4

Figure 1. shows the results of SDS-PAGE (15%), which determined the molecular weight of proteins of *Naja Naja *venom using the protein ladder (14, 20, 21.2, 22, 25, 32, 34, 37, 50, 65, 75, and 150 kDa).

*Western Blot Analysis*

**Figure 2:**
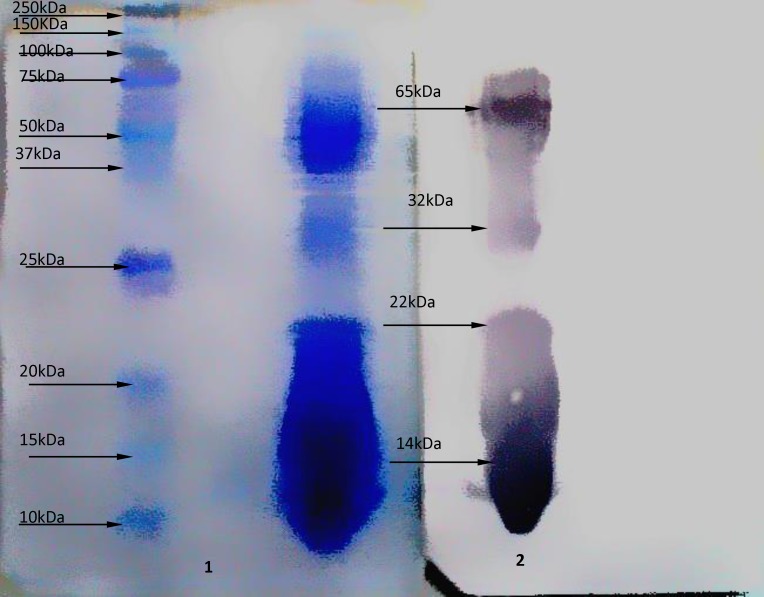
Western blot analysis on *Naja Naja *venom .1) Molecular weight markers and crude venom from *Naja Naja *snake after transfered onto nitrocellulose membrane, by SDS-PAGE (15%). Coomassie brilliant blue R-250 stain 2) Immunological reactivity of antibody and crude venom from* Naja Naja* snake

Western blot analysis on antigen at the dilution 1:20 with serum hyper- immunized- horse, Figure (2) shows 4 sharp bands as (14, 22, 32, and 65 kDa) molecular weights.

*Evaluation of Isolation of 14, 22, 32, and 65 kDa immunodominant proteins After Electro-Elution*

The Electro- elution method was used for isolation of 14, 22, 32, and 65 kDa immunodominant proteins. Electro-elution of proteins from gel is the method used to recover proteins resolved by electrophoresis in polyacrylamide gel by transferring the protein molecules out of gel by means of an electric field (18, 19). Only 4 protein bonds with 14, 22, 32, and 65 kDa molecular weights were manifested in the coomassie staining gel of isolated proteins achieved from Electro-elution. Then, the concentrations of isolated proteins (14, 22, 32, and 65 kDa) were determined respectively as 1.3, 0.7, 0.7, and 1 mg/mL by the Lowry assay.

Immunodominant proteins were homogeneous as judged by SDS-PAGE, and respectively, with molecular mass of Acidic phospholipase A2 4 (14,015 Da with AC.no:Q6T179 and 16,013 kDa , AC.no: P00598), Cytochrome C oxidase subunit 1(COI) (22,915 Da ,AC.no: A0A0A7PA27), Snake venom serine protease (Nasp) (31,137 Da , AC.no A8QL53), Zinc metalloproteinase-disintegrin-like (K-like and atragin) (66,292 Da with AC.no D3TTC1 and 69,180Da ,AC.no D3TTC2) in Uniprot protein database (NCBI research) in agreement with previous reports (20, 21) .

*Disc Diffusion*

Table 1. shows antibacterial activity of four crude isolated immunodominant proteins of *N. naja* *Oxiana* (14, 22, 32 and 65 kDa) and also different antibiotics, against Gram-negative bacteria including *E.coli* and *Pseudomonas aeruginosa* and Gram-positive bacteria including *Bacillus subtilis* and *Staphylococcus aureus* on MH agar plate which produced inhibitory zones. These are important for the defensive mechanism of innate immunity system.

**Table 1:** Antibacterial activity of immunodominant proteins crude isolated of *N.naja oxiana* venom tested by disc-diffusion and compared to some standard antibiotics. Each number is presented as mean + SD of inhibition zone in mm,(n = 4

**Microorganism** **Antibiotics / Venom**	***Pseudomonas aeruginosa***	***E.coli***	***B. subtilis***	***S. aureuss***
Immunodominant protein 0f 14 kDa	10/83 ± 0/28	10/66 ± 0/28	16/5 ± 0/5	13/16 ± 0/28
Immunoominant protein of 22 kDa	6.7 + 0.64	7/1 ± 0/36	7/1 ± 0/36	10/13 ± 0/32
Immunodominant protein of 32 kDa	6/93 ± 0/11	7/6 ± 0/52	8/1 ± 0/36	12/73 ± 0/25
Immunodominant protein of 65 kDa	7/6 ± 0/52	7/43 ± 0/40	15/76 ± 0/25	14/5 ± 0/5
Ampicillin	0.0 + 0.0	8/26 ± 0/55	19/26 ± 0/64	24/13 ± 0/41
Gentamicin	18 ± 0/2	19/04 ± 0/16	35/88 ± 0/34	16/13 ± 0/20
Penicillin	0.0 + 0.0	0.0 + 0.0	9/13 ± 0/32	0.0 + 0.0
Ciprofloxacin	0.0 + 0.0	35/66 ± 0/57	30/88 ± 0/34	24/02 ± 0/13
Amoxiclav	0.0 + 0.0	14/27 ± 0/26	18/46 ± /57	20 ± 0/8

**Table 2:** Antibacterial activities of isolated immunodominant proteins of *Naja Naja Oxiana*

**Bacteria**	**Immunodominant Protein name/Standard Antibiotics**	**MIC(μg/mL)**
**Gram-negative**		
*Escherichia Coli*	Immunodominant protein 0f 14 kDa	75
	Immunoominant protein of 22 kDa	**>**190
	Immunodominant protein of 32 kDa	>175
	Immunodominant protein of 65 kDa	≥125
	Ampicillin	120
	Gentamicin	<10
	Penicillin	>200
	Ciprofloxacin	<5
	Amoxiclav	25
*Pseudomonas aeruginosa*	Immunodominant protein 0f 14 kDa	75
	Immunoominant protein of 22 kDa	**>**190
	Immunodominant protein of 32 kDa	>175
	Immunodominant protein of 65 kDa	≥125
	Ampicillin	>200
	Gentamicin	<10
	Penicillin	>200
	Ciprofloxacin	>200
	Amoxiclav	>200
*Staphylococcus aureus*	Immunodominant protein 0f 14 kDa	37.5
	Immunoominant protein of 22 kDa	**>**190
	Immunodominant protein of 32 kDa	87
	Immunodominant protein of 65 kDa	26
	Ampicillin	25
	Gentamicin	10
	Penicillin	>200
	Ciprofloxacin	<5
	Amoxiclav	10
*Bacillus subtilis*	Immunodominant protein 0f 14kDa	37.5
	Immunoominant protein of 22 kDa	**>**190
	Immunodominant protein of 32kDa	>175
	Immunodominant protein of 65 kDa	31
	Ampicillin	25
	Gentamicin	10
	Penicillin	120
	Ciprofloxacin	<5
	Amoxiclav	25

*Broth Microdilution Assay of Four Identified Immunodominant Proteins*

The MIC values of these immunodominant proteins, also of standard antibiotics, were calculated against four bacteria. Table 2. shows these results.

## Discussion

Previous studies have demonstrated that a variety of snake venoms have antibacterial effects and are highly active in killing a wide range of bacteria (14-16). Generally, antibacterial proteins are cationic in nature (22) and it is believed that they exert their bactericidal effect through permeabilization of bacterial membranes via a thinning process (23, 24) that involves the destabilization of the membrane bilayer (25). On the other hand, bactericidal mechanism used by anionic antimicrobial proteins against Gram-positive bacteria such as *S. aureus *obviously involves interactions with the membrane lipid head group region (24). In addition to membrane permeabilization, antibacterial proteins, and peptides kill bacteria by inhibition of macromolecular biosynthesis (26) and interacting with specific basic components inside the bacteria (27). Some studies have suggested that induction of autolytic enzymes by peptides and proteins may be associated with bacterial cell death (28). The mechanisms of bactericidal and membrane damaging effects were confirmed ultrastructrually (29). Previous studies have reported a proteinase from *Agkistrodon halys *venom of viper family that inhibits the growth of *S.aureus *(30) and other drug-resistant human pathogens (31). Reported the MICs of basic myotoxic PLA2 (EcTx-I) from *Echis carinatus *venom was (15g/mL) against *Burkholderia pseudomallei *(KHW) and 30 g/mL against *Enterobacter aerogenes. *Also, MIC of *Daboia russelli russelli *(DRR-PLA2) against *S. aureus* was reported to be 62.3 g/mL (21) and for a viper metalloprotein at comparatively lower concentrations against *S. aureus *(MIC, 7.5 μM ) (31). Also antibacterial effects of L-amino acid oxidase in the venom of Cobra and Viper have been investigated against Gram-positive and Gram-negative bacteria. This enzyme with high molecular weight has been proved to have a strong antibacterial effect (4). Mui Li Lee *et.al* , 2011 (32) isolated LAAO (L-amino acid oxidase ) 65 kDa from the venom of *King Cobra (Ophiophagus hannah) *and have demonstrated that LAAO has higher antibacterial effect against Gram-positive bacteria compared to Gram-negative bacteria. In contrast, galactose binding lectin (33), phospholipase A2 (4, 21, 34), acuthrobin-C (35), onwaprin, and 50-amino acid cationic protein (35)that were extracted from snake venoms were low molecular weight proteins (10-17 KDa) with strong antibacterial activities. In addition, Hsuan-Wei Hung *et al*., 2015 (20), have reported the molecular weight of CTX_s_ (8980-9323 Da), acidic phospholipase A_2 _(PLA_2__s_ 16,013 Da) and Zinc metalloproteinase-disintegrin-like (*K-like*) and *atragin* (SVMP 66,292 Da and 69,180 Da), respectively, extracted from *Naja atra* with high immunogenicity and also high immunoreactivity to immunize mice against snakebite of individual *Naja atra *snake venom. But they did not investigate the antibacterial effects of these proteins. Most authors have not compared the antibacterial activity of the immunodominant proteins with common antibiotics, as well as their MICs against Gram-positive and Gram-negative bacteria. Hence, in the present study, cobra (*Naja Naja Oxiana*), the only major species of snakes found in the North-east of Iran, was chosen and immunogenicity and antibacterial effects of its immunodominant proteins were investigated. The MICs of immunodominant protein of 14kDa against *S. aureus and B. subtilis* were 37.5 µg/mL. The MICs against *E.coli* and *P.aeruginosa *were 75µg/mL. The MICs of immunodominant protein of 22kDa against *S. aureus, B. subtilis, E.coli, and P. aeruginosa *were >190 µg/mL. The MICs of immunodominant protein of 32 kDa against *E.coli, P. aeruginosa* and *B. subtilis* were >175µg/Ml and against *S. aureus *it was 85µg/mL. The MICs of immunodominant protein of 65 kDa against* E.coli *and* P.aeruginosa *were ≥125 µg/mL and against *S. aureus *and* B. subtilis* the MICs were 26 µg/mL and 31 µg/mL, respectively. The MICs of immunodominant proteins of 14 kDa and 65 kDa against the tow Gram-positive bacteria were comparable to the antibiotics tested, except for Ciprofloxacin, which was far more potent than the immunodominant proteins. Also the results of disc diffusion showed that the antibacterial effect of immunodominant protein of 14 kDa against *P. aeruginosa* was more than Ampicillin, Penicillin, Ciprofloxacin, and Amoxiclav, and against *E.coli* it was more than Ampicillin and penicillin, and almost the same as Amoxiclav. Antibacterial effect of immunodominant protein of 14 kDa against *B. subtilis* was more than penicillin and almost the same as Ampicillin and Amoxiclav. Also antibacterial effect against *S. aureus* was almost the same as Gentamicin. Antibacterial effects of immunodominant proteins of 22 kDa and 32 kDa against *S. aureus *was more than penicillin. Antibacterial effect of immunodominant protein of 65 kDa against* B. subtilis* was almost the same as Ampicillin and Amoxiclav; the effect was almost the same as Gentamicin and more effective than Penicillin against *S. aureus.* Our results on antibacterial activities of these proteins with small molecular weights ≤ 65 kDa, firmly confirmed the findings of previous studies that immunodominant proteins of 14, and 65 kDa, with high immunogenicity and also high immunoreactivity were very effective in inhibiting the two Gram-positive bacteria (*S.aureus, B.sub*). However these proteins were only slightly effective against the two Gram-negative bacteria of *P.aeruginosa *and *E.coli*. Immunodominant proteins of 22 kDa and 32 kDa with high immunogenicity showed a slight antibacterial activity against the Gram-positive and Gram-negative bacteria in comparison with the other immunodominant proteins. Therefore, the proteins 22 kDa and 32 kDa that has high immunogenicity, cannot have high antibacterial activity. In addition, the results (Tables 1 & 2) showed that immunodominant proteins have more potent antimicrobial effects against the Gram-positive bacteria (*S.aureus, B.subtilis*) than the Gram-negative bacteria (*E.coli, P.aeruginosa*). We hope that this study will be useful in finding some alternative agents for drug-resistant microorganisms. 

## Conclusion

In the present study four proteins of high immunogenicity with molecular weights of 14, 22, 32, and 65 kDa were identified. Two novel antibacterial immunodominant proteins of 14 kDa and 65 kDa with high immunogenicity and high antibacterial activity against Gram-positive bacteria and with slightly antibacterial effect against Gram-negative bacteria (see Table 1 and 2) and two novel immunodominant proteins of 22 kDa and 32 kDa, with high immunogenicity but slight antibacterial activity against the Gram-positive bacteria and Gram-negative bacteria were identified.

It was shown that the antibacterial activities of immunodominant proteins of 14 kDa and 65 kDa were comparable to Penicillin and Gentamicin against *S.aureus*. The effects were comparable to penicillin, Ampicillin, and Amoxiclav against *B.subtilis.*

This is a simple and economic method for preparing suitable antibacterial immundominant proteins from individual venom components.
